# Edinburgh Surgical Hospital

**Published:** 1833-06

**Authors:** 


### EDINBURGH SURGICAL HOSPITAL.

*Ligature of the Carotid Artery, on account of Hemorrhage from the Ear and Fauces.* [*From* Mr. Symes' *Report; Edinburgh Med. and Surg. Journal.*]

Though this case was not treated in the hospital, it deserves to be related, from the unusual circumstances with which it was attended. I am indebted to Mr. Cheyne, of Leith, for the following history of it:

William Mason, a delicate boy, nine years of age, complained, in the latter end of August 1832, of a soreness in the left fauces. This ceased in two days. About a week afterwards, viz. on the 29th August, he was seized with pain and swelling of the right fauces and smart accompanying fever. The fever left him in a day or two, but there remained a painful external swelling situated between the upper part of the larynx and the mastoid muscle. This increased gradually, extending upwards to the jaw, and finally to the tube of the external ear.

On the night of the 8th September, a discharge of pus took place from the meatus externus of the ear, and it continued till the following evening. A teacupful, probably, was the quantity altogether discharged. The latter portions were tinged with blood. Soon after the discharge of pus had ceased, the ear having been lightly bound up, the boy raised an alarm, in consequence of a flow of blood from the ear. Upon removing the bandage, it continued profuse for a short time. It then ceased. Some ounces appeared to have been lost. A compress and bandage were applied. The swelling dependent on the abscess had disappeared entirely.

13th. A fresh bleeding from the ear. Sponge introduced with compress and bandage over it. Since the 9th, (the date of the first bleeding,) a swelling has been formed between the angle of the jaw, mastoid process, and external tube of the ear; it has no pulsation.

16th. The swelling considerably increased; it now extends downwards, bordering on the larynx. On examining the mouth, a swelling is perceived like that produced by an enlarged tonsil pushing forward the anterior curtain of the palate, and insinuating itself between the jaw and lining membrane of the mouth; it is rather pale in colour, soft, and elastic. The following symptoms are present: considerable difficulty of breathing and swallowing, imperfect articulation, frequent hawking of phlegm from the throat, difficulty of closing the jaws, difficulty of lying down, very uneasy expression of the countenance, with constant watering of the eyes. The pulse of the temporal artery to be distinctly felt.

18th. In the evening he discharged suddenly from the mouth two or three ounces of florid blood. For a day or two before this the phlegm from his throat had been tinged with blood. The case appearing to be one of extreme hazard, and the object of present apprehension, hemorrhage, likely to be fatal, either by its quantity or through the vicinity of the trachea, by causing suffocation, it was suggested by Dr. Combe to tie the common carotid artery, and this operation was proposed to the parents. They, however, did not accede to it at that time. It was then agreed to request Mr. Syme's opinion on the case. Mr. S. having approved of the measure, it was again recommended (by him) to the parents, who now consented to it. The artery was then tied at the side of the larynx by Mr. Syme.

October 1st. Immediately after the operation an improvement in all respects took place, and has been gradually increasing till to-day. The operation itself gave rise to no inconvenience which would not have resulted from a simple wound of the parts. The external swelling decreased quickly, and is now gone. There still remains a slight internal swelling in the situation of the tonsil. No pulsation of the temporal artery. Since the first bleeding at the ear, there has always been an oozing from it of a thin bloody ichor.

On the evening of this day, while he was reading aloud, a profuse bleeding took place from the internal fauces, and at the same time from the ear. It soon ceased. About ten ounces were discharged in all. On examining the mouth, some clotted blood was seen adhering to the right tonsil. He had been allowed incautiously to go out of doors in the forenoon, and had run about without restraint.

2d. A tumor equal in size to the half of a hen's egg has been formed since last evening between the angle of the jaw, mastoid process, and tube of the ear. There is no increase of the swelling in the mouth.

6th. The external swelling decreased in some degree. There has been no discharge of the ichorous fluid from the ear since the 1st instant.

In the evening a fresh discharge of blood from the internal fauces and nostrils, (from one source, however, the right side of the fauces as it appeared.) It soon ceased, but not before about twenty ounces were lost. Immediately after he had syncope. In two hours he had rallied, and was asleep with a pulse at 120.

8th. In the evening, a bleeding from the fauces to the amount of eighteen ounces, followed by faintness and very small pulse.

After the 8th there was no return of bleeding, the swelling decreased, and he gradually recovered. For some time the right tonsil appeared of a dark colour.

He is now as well as before the commencement of his illness.

*Wry-Neck, depending on Contraction of the Sterno-Mastoid, remedied by division of the Muscle.* [*Ibid.*]

Matthew Cullen, æt. six, Dunbar, admitted November 2, 1832. The head was much inclined to the left side, and could not be elevated, owing to the rigid contraction of the left sterno-mastoid, the sternal part of which felt like a tense cord. The complaint had existed upwards of twelve months, and had resisted blisters, with other similar means of remedy. The introduction of needles having been tried without any benefit, it was thought necessary to divide the contracted part of the muscle. This was effected by entering a sharp-pointed narrow knife a little nearer the trachea than its sternal margin, about an inch above the clavicle, and then pressing the blade against the tense fibres. A sudden snap, which shook the patient's frame, was immediately perceived, and all trace of the contraction disappeared. The knife was withdrawn, and the small puncture occasioned by it in passing through the skin afforded the only perceptible indication of what had been done. No pain or other bad consequences followed, and the cure might be regarded as at once complete.

